# Effects of *OsRCA* Overexpression on Rubisco Activation State and Photosynthesis in Maize

**DOI:** 10.3390/plants12081614

**Published:** 2023-04-11

**Authors:** Yujiao Feng, Hao Wu, Huanhuan Liu, Yonghui He, Zhitong Yin

**Affiliations:** 1 Jiangsu Key Laboratory of Crop Genomics and Molecular Breeding/Key Laboratory of Plant Functional Genomics of the Ministry of Education/Jiangsu Key Laboratory of Crop Genetics and Physiology/Joint International Research Laboratory of Agriculture and Agri–Product Safety of the Ministry of Education, Agricultural College of Yangzhou University, Yangzhou 225009, China; 2 Jiangsu Co–Innovation Center for Modern Production Technology of Grain Crops, Yangzhou University, Yangzhou 225009, China

**Keywords:** energy conversion efficiencies in photosystems (PS) I and PSII, gas exchange, maize, photosynthesis, rubisco activase

## Abstract

Ribulose–1,5–bisphosphate carboxylase/oxygenase (Rubisco) is the rate–limiting enzyme for photosynthesis. Rubisco activase (RCA) can regulate the Rubisco activation state, influencing Rubisco activity and photosynthetic rate. We obtained transgenic maize plants that overproduced rice *RCA* (*OsRCA*^OE^) and evaluated photosynthesis in these plants by measuring gas exchange, energy conversion efficiencies in photosystem (PS) I and PSII, and Rubisco activity and activation state. The *OsRCA*^OE^ lines showed significantly higher initial Rubisco activity and activation state, net photosynthetic rate, and PSII photochemical quantum yield than wild–type plants. These results suggest that *OsRCA* overexpression can promote maize photosynthesis by increasing the Rubisco activation state.

## 1. Introduction

Photosynthesis is a complex physiological and biochemical process involving light and dark reactions [1]. Ribulose–1,5–bisphosphate carboxylase/oxygenase (Rubisco) is the key CO_2_–fixing enzyme in the photosynthetic dark reaction [2]. Rubisco carboxylation activity depends on Rubisco abundance and activation state in vivo. Few studies on improving the photosynthetic capacity of C4 plants have been conducted in comparison to C3 plants [3]. In C4 plants, because of a CO_2_–concentrating mechanism (CCM), CO_2_ accumulates around vascular bundle sheath cells and leads to a significantly lower Rubisco abundance than in C3 plants [4,5]. Recently, in the C4 plant maize (*Zea mays*), Rubisco abundance was increased through simultaneous overexpression of Rubisco and Rubisco assembly chaperone, but the improvement in photosynthetic efficiency was much less than the increase in Rubisco abundance [6]. The limited effect of increased Rubisco content on photosynthesis was related to a low Rubisco activation state [6]. Therefore, increasing the Rubisco activation state might help to improve photosynthetic efficiency [7].

Rubisco activase (RCA) regulates Rubisco activation by scavenging inhibitors such as sugar phosphate from the active site to maintain its high activation state [8,9]. Higher maize *RCA* (*ZmRCA*) expression in the filling stage results in greater photosynthetic capacity [10,11] and the expression levels of *RCA* genes are positively associated with grain yield in 123 maize inbred lines [11]. The correlation between the yield and expression of *RCA* is higher than that of three C4–specific genes associated with high–efficiency photosynthesis that is widely studied in maize [11]. Therefore, alteration of *RCA* expression levels might be a useful strategy to optimize Rubisco activation and improve photosynthetic capacity in maize.

Based on a dynamic photosynthesis model, it was suggested that increasing the activity of RCA or Rubisco can be an option to increase photosynthetic efficiency in C4 crops [12]. Down–regulation of RCA in the C4 plant Flaveria bidentis decreased Rubisco carbamylation and leaf photosynthesis [13]. Two *RCA* genes, an α–isoform ZmRCAα and a β–isoform ZmRCAβ, were identified in maize [11], while only one *RCA* gene, *OsRCA*, which encodes both α and β isoforms of RCA by alternative splicing of RCA transcripts, was identified in rice (*Oryza sativa*) [14]. Although sequence alignment revealed a high similarity in RCA amino acid between maize and rice, there were differences in their Rubisco recognition domain [15]. Overexpressed foreign *RCA* from closely related species in rice can enhance the Rubisco activation state [15]. These findings prompt us to ask whether overexpression of *OsRCA* would increase maize Rubisco’s activation state and photosynthetic capacity. In this study, we overexpressed *OsRCA* in maize and measured Rubisco activity, Rubisco content, Rubisco activation state, gas exchange parameters, and energy conversion efficiencies in photosystems (PS) I and PSII. Compared to WT, maize lines overexpressing *OsRCA* showed a substantial increase in Rubisco activation state and photosynthetic capacity while maintaining a similar level of Rubisco content. Our results might provide a way to breed maize cultivars with high photosynthesis.

## 2. Results

### 2.1. Molecular Characterization of Transgenic Plants

**Figure 1:**
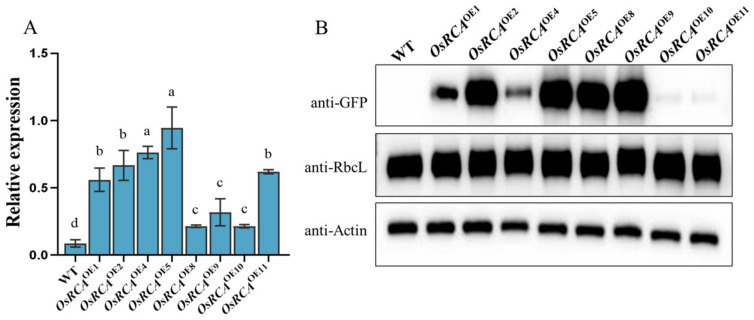
Molecular characterization of WT and OsRCA^OE^ lines. (**A**) OsRCA mRNA abundance. The different letters indicate significant differences at *p* < 0.05. (**B**) OsRCA–GFP and RbcL protein expression. Anti–actin acted as an internal control to correct protein quantification. The fusion protein and RbcL protein were detected with Western blotting using anti–GFP and anti–RbcL.

In order to obtain maize lines overexpressing *OsRCA* (*OsRCA*^OE^), we introduced *OsRCA* fused with a green fluorescent protein (*GFP*) into maize inbred line B104 driven by the 35S promoter. Eight independent *OsRCA*^OE^ lines were obtained for real–time PCR (qRT–PCR) and Western blot analysis. qRT–PCR indicated that the *OsRCA* expression levels increased 2.5– to 11–fold in *OsRCA*^OE^ lines compared to the WT (B104; Figure 1A). Accordingly, Western blots probed with a GFP antiserum showed that the amount of OsRCA–GFP protein was increased in the *OsRCA*^OE^ lines (Figure 1B). Considering their transcript and protein levels, we selected three representative lines (*OsRCA*^OE2^, *OsRCA*^OE5^, and *OsRCA*^OE9^) for subsequent studies on photosynthetic characteristics.

### 2.2. Rubisco Activity, Content, and Activation State in Transgenic Lines

**Figure 2:**
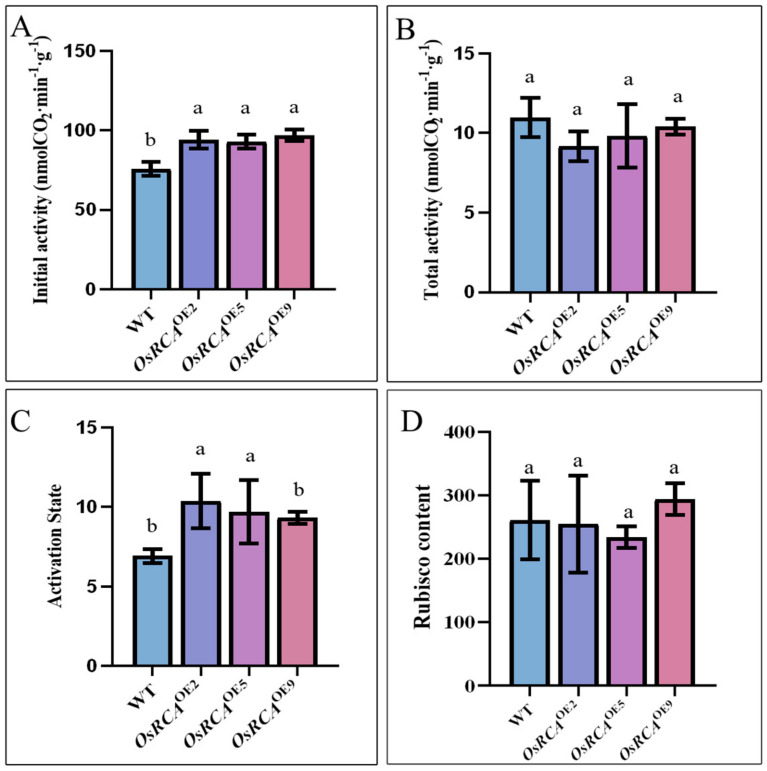
Rubisco activity and content in WT and *OsRCA*^OE^ lines during maize grain filling. (**A**) Initial Rubisco activity. (**B**) Total Rubisco activity. (**C**) Rubisco activation state; the formula is initial Rubisco activity/total Rubisco activity. (**D**) Rubisco content by ELISA. The LSD test was used for multiple data comparisons. The different letters indicate significant differences at *p* < 0.05.

In order to determine whether overexpression of *OsRCA* affected Rubisco activity and activation state in maize, we measured the initial and total activities in the WT and *OsRCA*^OE^ lines. *OsRCA* overexpression in maize had no effect on the total activity of Rubisco (Figure 2B). However, the initial activity of Rubisco in *OsRCA*^OE2^, *OsRCA*^OE5^, and *OsRCA*^OE9^ was 24.17%, 22.67%, and 28.00%, respectively, higher than that in the WT (Figure 2A). The ratio of initial to total activity can be used to reflect the Rubisco activation state [16]. Consistently, the Rubisco activation state in *OsRCA*^OE2^ and *OsRCA*^OE5^ was 49.84% and 40.16%, respectively, higher than that in the WT (Figure 2C).

In order to investigate whether overexpression of *OsRCA* impacted Rubisco content, we determined the content of Rubisco by Western Blot analysis and Enzyme–Linked Immunosorbent Assay analysis (ELISA) (Figure 1B and Figure 2D). The ELISA results showed that the Rubisco content was not significantly different between the WT and *OsRCA*^OE^ lines (Figure 2D). In agreement with this, Western blots probed with Rubisco large subunit (RbcL) antibody also showed that the overexpression of *OsRCA* did not significantly affect the expression of Rubisco content (Figure 1B).

### 2.3. Gas Exchange Parameters of Transgenic Lines

In our previous study, *RCA* was expressed in leaves at a higher level at the grain–filling stage than at the other growth period [11]. To assess the effect of *OsRCA* overexpression on maize photosynthetic characteristics, we measured gas exchange parameters in the WT and *OsRCA*^OE^ lines at the grain–filling stage. As analyzed with analysis of variance (ANOVA), the data showed that *OsRCA* overexpression had significant effects on the net photosynthetic rate (Pn) and intercellular CO_2_ concentration (Ci) but not stomatal conductance or transpiration rate (Table 1). Pn was significantly higher in *OsRCA*^OE2^ and *OsRCA*^OE5^ (by 13.19% and 10.12%, respectively) than in the WT; however, Ci was 35.37% and 44.62% lower in *OsRCA*^OE2^ and *OsRCA*^OE5^, respectively, than in the WT (Table 1). We observed a similar trend for Pn and Ci in *OsRCA*^OE9^, although the differences between this line and WT were not statistically significant.

### 2.4. PSII and PSI Energy Conversion–Related Parameters in Transgenic Lines

We simultaneously measured PSII and PSI energy conversion–related parameters in the WT and *OsRCA*^OE^ lines with Dual–PAM–100. Three parameters related to PSII were recorded, including Y(II), Y(NPQ), and Y(NO). Y(II) represents the efficiency of photochemical energy conversion by PSII; Y(NPQ) and Y(NO) reflect the quantum yields of regulated and non–regulatory energy dissipation, respectively [17]. Three parameters related to PSI energy conversion were recorded, including PSI photochemical efficiency Y(I), the non–photochemical quantum yield of the PSI donor side Y(ND), and the non–photochemical quantum yield of the PSI acceptor side Y(NA) [17].

**Figure 3:**
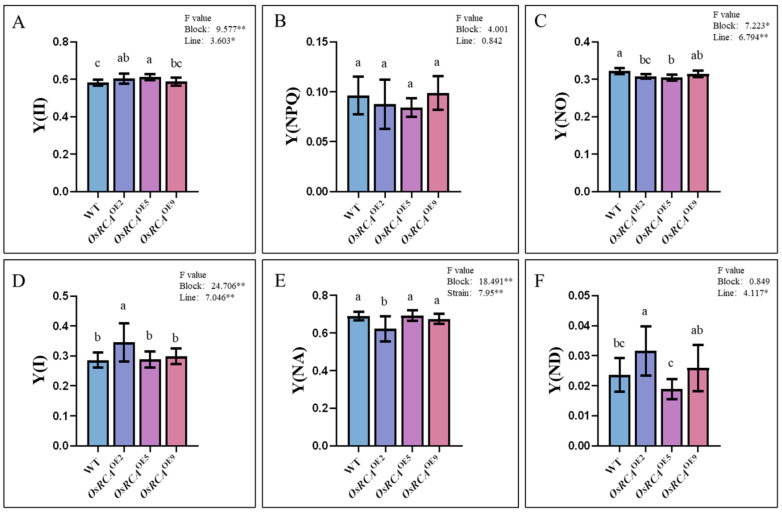
PSII and PSI energy conversion–related parameters of WT and *OsRCA*^OE^ lines. (**A**) Y(II), PSII photochemical energy conversion yield. (**B**) Y(NPQ), the quantum yield of regulatory energy dissipation at PSII. (**C**) Y(NO), quantum production of non–regulatory energy dissipation at PSII. (**D**) Y(I), PSI photochemical energy conversion yield. (**E**) Y(NA), the non–photochemical quantum yield of the PSI acceptor side. (**F**) Y(ND), the non–photochemical quantum yield of the PSI donor side. Different letters above the bars indicate significant differences between different lines at *p* < 0.05. * *p* < 0.05, ** *p* < 0.01.

Y(II) was 3.81% and 5.09%, significantly higher (Figure 3A) in *OsRCA*^OE2^ and *OsRCA*^OE5^ than in the WT, while Y(NO) was 4.32% and 5.44%, respectively, significantly lower (Figure 3C) compared to the WT. There were no obvious differences in Y(NPQ) between the WT and *OsRCA*^OE^ lines (Figure 3B). Y(I) and Y(ND) were significantly higher (Figure 3D,F), whereas the Y(NA) was significantly lower (Figure 3E) in *OsRCA*^OE2^, which had the highest Rubisco activation state (Figure 2C) than in the WT. The PSI parameters of *OsRCA*^OE5^ and *OsRCA*^OE9^ were not significantly different from those of the WT.

## 3. Discussion

In this study, we successfully overexpressed *OsRCA* in maize. qRT–PCR and Western Blot analysis showed that the three *OsRCA*^OE^ lines– *OsRCA*^OE2^, *OsRCA*^OE5^, and *OsRCA*^OE9^—expressed rice RCA at high levels (Figure 1). Rubisco is a key factor that limits photosynthetic carbon assimilation. Only Rubisco activated by *RCA* can catalyze CO_2_ fixation [18]; therefore, it is the amount of activated Rubisco, rather than the total amount of Rubisco, that affects the photosynthetic rate. In this study, *OsRCA* overexpression enhanced Rubisco’s initial activity and activation state (Figure 2A,C). This result is comparable to that of a previous study in which Rubisco activation was enhanced because of *RCA* overexpression in rice [19]. Subtle changes in RCA amino acids or domains can affect the interaction of RCA with Rubisco [20,21]. Replacing wheat RCA methionine located in the RCA AAA^+^ module with isoleucine improved the thermostability of RCA and enhanced the enzyme activity efficiency at a higher temperature [22]. According to a previous study, spinach RCA could not recognize tobacco Rubisco due to amino acid sequence differences in substrate recognition sites of the two species [15]. A comparison of the amino acid sequence showed that although rice and maize have a high similarity in RCA, they do show some differences. For example, an important amino acid that interacts with Rubisco is Arg in maize but Lys in rice (Figure S1). The increased Rubisco activation in maize by *OsRCA* overexpression in the present study suggests that the rice RCA can recognize and activate maize Rubisco. The total activity is proportional to Rubisco content [16]. In this study, compared to the WT, Rubisco’s total activity showed no significant difference (Figure 2B). In agreement with this observation, Western blot and ELISA analysis showed that *OsRCA* overexpression did not significantly affect the expression of Rubisco content (Figure 1B and Figure 2D). In a previous study, it was reported that *RCA* overproduction in rice affected Rubisco content negatively [23]. In contrast, other studies revealed that Rubisco content does not change in plants in which *RCA* is suppressed [24] and that *RCA* overproduction is possible without a reduction in Rubisco content [19], which is in agreement with our present study. The discrepancy may be attributable to the use of different crops or different growing environments.

In a previous study, Pn and electron transfer rates were enhanced in transgenic rice that overexpressed *OsRCA* [25]. *RCA*–overexpressing transgenic cucumber plants showed increased growth via the promotion of photosynthesis [26]. In this study, Pn was found to be higher in *OsRCA*^OE^ lines than in the WT (Table 1). The value of Ci is affected by the CO_2_ concentration of the air around the leaves, stomatal conductance, mesophyll conductance, and photosynthetic activity of mesophyll cells [27]. When the concentration of carbon dioxide in the air remains constant, changes in mesophyll cell photosynthetic activity and stomatal conductance produce changes in Ci [27]. Previous studies have found a negative correlation between Pn and Ci in the upper, middle, and lower parts of rice sword–leaf. This negative correlation was due to the higher activity or greater amount of activated Rubisco in the middle of the leaf resulting in a lower Ci [28]. In this study, Ci was lower in *OsRCA*^OE^ lines than in WT, with no significant differences in stomatal conductance and transpiration rate. These results indicate that the lower Ci in *OsRCA*^OE^ lines might be caused by an increased Rubisco activation state, which speeds up CO_2_ fixation rather than by stomatal factors (Table 1).

The measurement of PSII and PSI energy conversion–related parameters requires photosynthetic samples to reach a light–adapted steady state after illumination, where both light and dark reactions of photosynthesis are functional. Thus, similar to photosynthetic gas exchange parameters, these parameters are influenced by the intrinsic function and structure of the photosynthesis process light reaction apparatus and the biochemical or physiological processes in the photosynthetic dark reaction. RCA functions mainly in the photosynthetic dark reaction and is unlikely to affect the intrinsic function and structure of the apparatus of photosynthetic light reaction. Measurement of prompt chlorophyll a fluorescence (OJIP) transient signals during brief exposure of a dark–adapted leaf to light have been widely used to reflect the intrinsic function and structure of the photosynthetic light reaction apparatus [29,30]. We did not observe an obvious difference in the OJIP transient shapes and the derived JIP–test parameters (Figure S2). JIP–test parameters can describe primary events in the photosynthetic light reaction, such as the light energy absorbed by the reaction center, electron transport capacity, and processes related to energy dissipation [30]. The similar value of the JIP–test parameters between *OsRCA*^OE^ lines and the WT (Figure S2) suggests that the changes in photosynthetic exchange parameters and PSII and PSI energy conversion–related parameters of transgenic lines in the present study were not attributed to the intrinsic function of the photosynthetic light reaction apparatus.

Reactive oxygen species produced by electron transport can induce photoinhibition around PSII and PSI, and the recovery time of PSI after photoinhibition is longer than that of PSII [31]. In the present study, the increase in Y(II) (Figure 3A) and decrease in Y(NO) (Figure 3C) suggest that *OsRCA* overexpression enhanced the photochemical efficiency of PSII and photoprotection and inhibited passive energy dissipation in maize. No significant changes in Y(NPQ) were observed, indicating that the active energy dissipation of PSII was not affected (Figure 3B) [32]. The change in Y(II) caused by *OsRCA* overexpression was consistent with that in Pn. Given that the intrinsic function and structure of the photosynthetic light reaction apparatus were unaffected by *OsRCA* overexpression, we propose that the increase in Y(II) and Pn could be attributed to the enhanced Rubisco initial activity and activation state. Overproduction of *RCA* has been proven to make PSI susceptible to photoinhibition and decrease the robustness of PSI [31]. We did not obtain similar results in this study. The changes in Y(I) caused by *OsRCA* overexpression were not consistent among the three transgenic maize lines (Figure 3D); Y(I) significantly increased in *OsRCA*^OE2^ when compared with the WT but showed no obvious changes in the other two lines. Unlike PSII, which correlates well with linear electron flow along the photosynthetic electron chain, PSI is involved with both linear and cyclic electron flow. The latter could have affected the changes in PSI. In addition, the amount of RCA might also affect the function of PSI. We speculate that the function of PSI can be positively regulated only when *OsRCA* is overexpressed to a certain extent. Further studies are required to reveal the detailed mechanism involved in electron transport around PSI following *OsRCA* overexpression.

## 4. Materials and Methods

### 4.1. Cloning and Transformation of Rice RCA

Total RNA was extracted from rice leaves (*Oryza sativa* L. ssp. *japonica*) with TRIzol reagent (Vazyme Biotech Co., Ltd., Nanjing, China, R401–01). The first–strand cDNA was synthesized from the total RNA using the ABScript II cDNA First–Strand Synthesis Kit (ABclonal Technology Co., Ltd., Wuhan, China, RK20400). A 20 μL volume of reverse transcription reaction was set up as follows: 2 μL d(T)_23_VN, 1 μL dNTPs, 10 μL 2 × ABScript II Reaction Mix, 2 μL 10 × ABScript II Enzyme Mix, 1μg RNA and nuclease–free H_2_O up to 20 μL. The full–length cDNA of rice *RCA* (*OsRCA*, LOC4351224) was amplified using PCR with gene–specific primers (Table S1). *OsRCA* gene was connected to the vector pCAMBIA3301–GFP driven by the 35S promoter. The recombinant vector was introduced into maize inbred line B104 via *Agrobacterium*–mediated gene transfer. Seeds collected from self–crossing T_0_ maize progeny were used to propagate *OsRCA* overexpressed transgenic plants (Weimi Biotechnology Co., Ltd., Changzhou, China).

### 4.2. Plant Materials and Growth Conditions

The WT (B104) and *OsRCA*^OE^ lines were planted in a greenhouse at the College of Agriculture, Yangzhou University. The experiment was based on a randomized block design with two replications. Every plant was grown in a regular solar–greenhouse environment. At the grain–filling stage, ear leaves were selected to measure the Rubisco activity, Rubisco content, gas exchange, and PSII and PSI energy conversion–related parameters.

### 4.3. Real–Time PCR Assay

Total RNA was extracted from the WT and *OsRCA*^OE^ lines with TRIzol reagent (Vazyme Biotech Co., Ltd., Nanjing, China, R401–01). The cDNA was synthesized from 1 μg total RNA using the ABScript III RT Master Mix for qPCR (ABclonal Technology Co.,Ltd., Wuhan, China, RK20429). A 20 μL volume of reverse transcription reaction was set up as follows: 4 μL 5 × ABScript III RT Mix, 1 μL 20 × gDNA Remover Mix, 1 μg RNA, and nuclease–free H_2_O up to 20 μL. The reaction system was set on the PCR instrument as follows: 2 min at 37 °C, 15 min at 55 °C, and 5 min at 85 °C. These cDNAs were used for real–time PCR.

The relative mRNA expression of *OsRCA* in maize was analyzed using real–time PCR with the Universal SYBR Green Fast qPCR Mix (ABclonal Technology Co.,Ltd., Wuhan, China, RK21203) in accordance with the instructions of the manufacturer. Maize *GAPDH* (GRMZM2G046804) was used as an internal control. The primer sequences for *OsRCA* and *GAPDH* are provided in Table S2. The 20–μL reaction system was composed of 0.4 μL forward and reverse primers, 10 μL 2× Universal SYBR Green Fast qPCR Mix, 4 μL cDNA template, and 5.2 μL ddH_2_O; after mixing and centrifugation, real–time fluorescence was quantitatively detected. The expression level of each sample was calculated using the 2^−ΔCt^ method. Every sample was checked in triplicate.

### 4.4. Western–Blot Analysis of OsRCA

Total leaf soluble protein was extracted with protein loading. B104 was used as the negative control. Leaf tissues (0.1 g) from the WT and *OsRCA*^OE^ lines were ground into powder in liquid nitrogen and then homogenized in protein loading buffer containing 200 mM Tris–HCl (pH 8.0), 2% sodium dodecyl sulfate (SDS), 0.5% bromophenol blue (BPB), and 1% glycerol. Then, the resulting homogenate was incubated in a metal bath at 100 °C for 30 min and centrifuged in a microcentrifuge at 12,000 rpm at 4 °C for 15 min. The supernatants were collected as total leaf soluble protein extracts. The proteins were subjected to western blot.

SDS–PAGE with 10% polyacrylamide gel (Vazyme Biotech Co., Ltd., Nanjing, China, E303–01) was used to separate the total protein. The protein electrophoresis solution was prepared by dissolving 19.6 g glycine, 3.02 g Tris, 1 g SDS in ddH_2_O to a final volume of 1 L. Electrophoresis was performed at 100 V for 30 min and then 130 V for 1.5 h. After electrophoretic separation, proteins were transferred to polyvinylidene fluoride (PVDF) membranes by electroblotting. The 1 × Tris–Glycine Transfer Buffer was prepared by dissolving 3.03 g Tris and 18.8 g glycine in ddH_2_O to a final volume of 1 L. Then, the PVDF membranes were blocked with 5% (*w*/*v*) nonfat milk for 1.5 h at 25 °C. The 5% (*w*/*v*) nonfat milk was prepared by dissolving 5 g nonfat milk powder in Tris Buffered Saline Tween–20 (TBST) to a final volume of 100mL. The TBST contains 24.2 g Tris, 80 g NaCl, 11 mL HCl, 1 mL Tween–20, and ddH_2_O to a volume of 1 L. After the blocking, the membranes were probed with anti–GFP, anti–RbcL, and anti–actin (ABclonal Technology Co.,Ltd., Wuhan, China, AE012, A23203, and AC009) diluted 1:5000 in TBST with 5% (*w*/*v*) nonfat milk for 1.5 h at 25 °C. After washing five times, the membranes were incubated in the corresponding secondary antibody for 1 h at 25 °C. The secondary antibodies of Goat anti–mouse IgG (AS003) and Goat anti–rabbit IgG (AS029) were purchased from ABclonal.

### 4.5. Rubisco Protein Quantification by ELISA

Leaf tissues (0.1 g) from *OsRCA*^OE^ and WT lines were ground into powder in liquid nitrogen, and the crude protein was extracted in 1 mL precooled enzyme extract buffer containing 50 mM Hepes–KOH (pH 7.5), 10 mM MgCl_2_, 1 mM EDTA, 20% (*v*/*v*) glycerin, 0.25% (*w*/*v*) bovine serum albumin, 1% (*v*/*v*) Triton–X100, 1 mM PMSF, and 0.5 mM DTT. The crude protein homogenate was centrifuged in a microcentrifuge at 12,000 rpm at 4 °C for 15 min. The content of the Rubisco in the crude protein supernatant was determined using Rubisco ELISA Kit Plant (Jiwei Biotechnology Co., Ltd., Shanghai, China, JW.PL1161). Standard and diluted samples were added according to the instructions. After that, horseradish peroxidase–conjugated monoclonal antibody against Rubisco was added and incubated at 37 °C for 1 h. After washing the plates five times with washing liquid, substrate A and B were added and incubated at 37 °C for 15 min in the dark. Quantities of Rubisco were calculated according to standard curves plotted by parallel assays using serial dilutions of standard samples.

### 4.6. Measurement of Gas Exchange

A CIRAS–3 portable gas exchange system (PP–Systems, Haverhill, MA, USA) was used to measure the gas exchange parameters in our laboratory in accordance with previously described methods [33]. The following conditions were maintained: CO_2_ concentration (390 μmol mol^−1^), air humidity (60%), PARi (1800 µmol m^−2^s^−1^), gas flow (100 cc min^−1^), and leaf temperature (25 °C) [33]. One week after pollination, the middle and upper regions of ear leaves were used for measurement. Each measurement was performed on the same cloudless sunny morning (8:30–11:30 a.m.). Three plants were selected for measurement in each line of each replication. The average data of the three plants represent the value of each line.

### 4.7. Rubisco Activity and Activation State in Leaf Extracts

After gas exchange parameter measurement, the ear leaves were rapidly frozen in liquid nitrogen and stored at −80 °C until used for Rubisco activity assay. The initial and total activity of Rubisco were determined in accordance with the instructions of the Rubisco enzyme activity assay kit (Grace Biotechnology Co., Ltd., Suzhou, China, G0602W48). Leaf tissues (0.1 g) from *OsRCA*^OE^ and WT lines were ground into powder in liquid nitrogen and homogenized in 1 mL precooled enzyme extract buffer. The resulting homogenate was centrifuged in a microcentrifuge at 12,000 rpm at 4 °C for 10 min. The supernatant and Reagents 1 through 5 were then added in turn to a 96–well plate according to the instruction at room temperature (25 °C). The 96–well plate containing the mix was placed into an enzyme–label instrument (Molecular Devices, Silicon Valley, SpectraMax^®^ 190) to measure Rubisco activity. During the measurement, the absorbance at 340 nm was recorded every 20 s, and a period of linear decline was selected for calculation. A fixed 1 nmol CO_2_ g tissue^−1^ min^−1^ is defined as one unit of enzyme activity based on the sample’s fresh weight. The ratio of initial activity to total activity was used to express the Rubisco activation state [16].

### 4.8. Measurement of PSII and PSI Energy Conversion–Related Parameters

As described by previous methods in our laboratory [17] and instrument instructions, a Dual–PAM–100 (Heinz Walz, Effeltrich, Germany) was applied to record the energy conversion efficiencies in PSI and PSII. Leaf samples were collected from the central part of ear leaves and wrapped with wet gauze to keep them wet for an hour of dark adaption. The P700+Fluo model was used to measure the parameters of the dark–adapted ear leaves. The minimal fluorescence (Fo) was obtained by suddenly applying measurement light. The maximum fluorescence (Fm) and P700 changes (Pm) were obtained by applying a saturation pulse (SP). Then, the actinic light was turned on, and SP was given simultaneously every 20 s to achieve a steady state. During this process, the parameters related to energy conversion efficiencies in PSI and PSII were calculated and recorded [34] (Table 2). Three plants were selected for measurement in each line of each replication. The average data of the three plants represent the value of each line.

### 4.9. Statistical Analysis

The data were analyzed using the statistical software package SPSS Statistics 17.0 for Windows (SPSS, Inc., Chicago, IL, USA). Single–factor ANOVA was used to compare lines and blocks. The least significant difference (LSD) test was performed at *p* < 0.05. Values represent the mean ± standard deviation (SD) of two replicates.

## 5. Conclusions

In this study, we introduced the 35S promoter driving *OsRCA*–*GFP* into maize and developed eight independent maize transgenic lines. To investigate whether the *OsRCA* overexpression would increase the maize Rubisco activation state and photosynthetic capacity under normal growth conditions, three representative transgenic lines with relatively high levels of *OsRCA* expression were grown in a regular solar–greenhouse environment. They were assessed for Rubisco activity, Rubisco content, Rubisco activation state, gas exchange parameters, and energy conversion efficiencies in PSI and PSII. The results showed that *OsRCA* overexpression enhanced Rubisco’s initial activity and activation state without affecting Rubisco content and consequently improved Y(II) and Pn substantially in maize. Therefore, increasing the Rubisco activation state through *RCA* overproduction could be an efficient strategy to improve photosynthetic capacity in maize under normal growth conditions. Several studies have proven that RCA can be more important in regulating plant photosynthesis under adverse environmental conditions [21,35]. It will be intriguing to investigate the effect of *OsRCA* expression on maize Rubisco activity and photosynthesis under multiple stress conditions, such as high temperature and different light regimes.

## Figures and Tables

**Table 1:** Gas exchange parameters at the grain–filling stage and ANOVA of WT and *OsRCA*^OE^ lines.

Lines	Net Photosynthetic Rate (μmol·m^−2^·s^−1^)	Intercellular CO_2_ Concentration	Stomatal Conductance (mol·m^−2^·s^−1^)	Transportation Rate (m mol·m^−2^·s^−1^)
WT	29.694 ± 4.490c	47.500 ± 11.776a	163.125 ± 27.217a	5.124 ± 0.580a
*OsRCA* ^OE2^	33.610 ± 4.962a	30.700 ±1 4.407bc	175.600 ± 26.222a	5.0520 ± 0.491a
*OsRCA* ^OE5^	32.700 ± 3.945ab	26.308 ± 16.373c	167.846 ± 19.196a	4.861 ± 0.333a
*OsRCA* ^OE9^	30.120 ± 4.380bc	37.933 ± 13.172ab	160.333 ± 27.168a	4.937 ± 0.448a
F value				
Block	34.926 **	1.448	28.217 **	0.840
Line	5.258 **	6.453 **	2.220	0.925

Note: Multiple comparisons of data were performed using the LSD test. The different letters indicate significant differences at *p* < 0.05. ** *p* < 0.01.

**Table 2:** Parameters related to energy conversion efficiencies in PSI and PSII.

Fluorescence parameter	Description
Y(II) = (Fm’ − Fs’)/Fm’	PSII photochemical energy conversion yield
Y(NPQ) = Fs’/Fm’ − Fs’/Fm	Quantum yield of regulatory energy dissipation at PSII
Y(NO) = Fs’/Fm	Quantum production of non–regulatory energy dissipation at PSII
Y(ND) = 1 − P700red	Non–photochemical quantum yield of the PSI donor side
Y(NA) = (Pm − Pm’)/Pm	Non–photochemical quantum yield of the PSI acceptor side
Y(I) = 1 − Y(ND) − Y(NA)	Photochemical efficiency of PSI

## Data Availability

Data will be made available on request.

## Supplementary Materials

The following supporting information can be downloaded at: https://www.mdpi.com/article/10.3390/plants12081614/s1, Figure S1. Alignment of the amino acid sequence of Rubisco activase in maize and rice; Figure S2: OJIP in WT and *OsRCA*^OE^ lines. (A) Original OJIP curve. Signals are plotted using a logarithmic time scale. (B) O–P standardized curve; the formula is W_OP_ = (F_t_ – F_O_)/ (F_P_ – F_O_). Signals are plotted using a logarithmic time scale. (C) Parameters deduced from JIP test method of WT and *OsRCA*^OE^ lines.; Table S1: Primer sequences for PCR; Table S2: Primer sequences for qRT–PCR.

## References

[1] Nelson N., Ben-Shem A. (2004). The complex architecture of oxygenic photosynthesis. Nat. Rev. Mol. Cell Biol..

[2] von Caemmerer S., Evans J.R. (2010). Enhancing C3 photosynthesis. Plant Physiol..

[3] Zhao H., Wang Y., Lyu M.A., Zhu X. (2022). Two major metabolic factors for an efficient NADP–malic enzyme type C4 photosynthesis. Plant Physiol..

[4] P’yankov V., Voznesenskaya E., Kondratschuk A., Black C. (1997). A comparative anatomical and biochemical analysis in *salsola* (Chenopodiaceae) species with and without a Kranz type leaf anatomy: A possible reversion of C4 to C3 photosynthesis. Am. J. Bot..

[5] Xu J., Bräutigam A., Weber A.P., Zhu X.G. (2016). Systems analysis of cis–regulatory motifs in C4 photosynthesis genes using maize and rice leaf transcriptomic data during a process of de–etiolation. J. Exp. Bot..

[6] Salesse-Smith C.E., Sharwood R.E., Busch F.A., Kromdijk J., Bardal V., Stern D.B. (2018). Overexpression of Rubisco subunits with RAF1 increases Rubisco content in maize. Nat. Plants.

[7] Carmo-Silva E., Scales J.C., Madgwick P.J., Parry M.A. (2015). Optimizing Rubisco and its regulation for greater resource use efficiency. Plant Cell Environ..

[8] Seemann J.R., Kirschbaum M.U., Sharkey T.D., Pearcy R.W. (1988). Regulation of Ribulose–1,5–bisphosphate carboxylase activity in *Alocasia macrorrhiza* in response to step changes in irradiance. Plant Physiol..

[9] Chao M., Yin Z., Hao D., Zhang J., Song H., Ning A., Xu X., Yu D. (2014). Variation in Rubisco activase (*RCAβ*) gene promoters and expression in soybean [*Glycine max* (L.) Merr]. J. Exp. Bot..

[10] Martínez-Barajas E., Molina-Galán J.D., Jiménez E.S. (1997). Regulation of Rubisco activity during grain–fill in maize: Possible role of Rubisco activase. J. Agric. Sci..

[11] Yin Z., Zhang Z., Deng D., Chao M., Gao Q., Wang Y., Yang Z., Bian Y., Hao D., Xu C. (2014). Characterization of Rubisco activase genes in maize: An α–isoform gene functions alongside a β–isoform gene. Plant Physiol..

[12] Wang Y., Chan K.X., Long S.P. (2021). Towards a dynamic photosynthesis model to guide yield improvement in C4 crops. Plant J..

[13] von Caemmerer S., Hendrickson L., Quinn V., Vella N., Millgate A.G., Furbank R.T. (2005). Reductions of Rubisco activase by antisense RNA in the C4 plant Flaveria bidentis reduces Rubisco carbamylation and leaf photosynthesis. Plant Physiol..

[14] To K.Y., Suen D.F., Chen S.C. (1999). Molecular characterization of ribulose–1,5–bisphosphate carboxylase/oxygenase activase in rice leaves. Planta.

[15] Fukayama H., Ueguchi C., Nishikawa K., Katoh N., Ishikawa C., Masumoto C., Hatanaka T., Misoo S. (2012). Overexpression of rubisco activase decreases the photosynthetic CO_2_ assimilation rate by reducing rubisco content in rice leaves. Plant Cell Physiol..

[16] Aluru M.R., Stessman D.J., Spalding M.H., Rodermel S.R. (2007). Alterations in photosynthesis in Arabidopsis lacking IMMUTANS, a chloroplast terminal oxidase. Photosyn. Res..

[17] Zhou R., Kan X., Chen J., Hua H., Li Y., Ren J., Feng K., Liu H., Deng D., Yin Z. (2019). Drought–induced changes in photosynthetic electron transport in maize probed by prompt fluorescence, delayed fluorescence, P700 and cyclic electron flow signals. Environ. Exp. Bot..

[18] Singh J., Pandey P., James D., Chandrasekhar K., Achary V.M., Kaul T., Tripathy B.C., Reddy M.K. (2014). Enhancing C3 photosynthesis: An outlook on feasible interventions for crop improvement. Plant Biotechnol. J..

[19] Suganami M., Suzuki Y., Kondo E., Nishida S., Konno S., Makino A. (2020). Effects of overproduction of Rubisco activase on Rubisco content in transgenic rice grown at different N levels. Int. J. Mol. Sci..

[20] Nagarajan R., Gill K.S. (2018). Evolution of Rubisco activase gene in plants. Plant Mol. Biol..

[21] Salvucci M.E. (2007). Association of Rubisco activase with chaperonin–60β: A possible mechanism for protecting photosynthesis during heat stress. J. Exp. Bot..

[22] Degen G.E., Worrall D., Carmo-Silva E. (2020). An isoleucine residue acts as a thermal and regulatory switch in wheat Rubisco activase. Plant J..

[23] Fukayama H., Mizumoto A., Ueguchi C., Katsunuma J., Morita R., Sasayama D., Hatanaka T., Azuma T. (2018). Expression level of Rubisco activase negatively correlates with Rubisco content in transgenic rice. Photosyn. Res..

[24] Eckardt N.A., Snyder G.W., Portis A.R., Orgen W.L. (1997). Growth and photosynthesis under high and low irradiance of Arabidopsis thaliana antisense mutants with reduced ribulose–1,5–bisphosphate carboxylase/oxygenase activase content. Plant Physiol..

[25] Wu H.R. (2004). Photosynthetic Characterizations of Transgenic Rice with Rca Gene Encoding the Large Isoformof Rice Rubisco Activase. Ph.D. Thesis.

[26] Bi H., Liu P., Jiang Z., Ai X. (2017). Overexpression of the rubisco activase gene improves growth and low temperature and weak light tolerance in *Cucumis sativus*. Physiol. Plant.

[27] Chen G.Y., Chen J., Xu D.Q. (2010). Thinking about the relationship between net photosynthetic rate and intercellular CO_2_ concentration. Plant Physiol. J..

[28] Zhang D., Wang X., Chen Y., Xu D. (2005). Determinant of photosynthetic capacity in rice leaves under ambient air conditions. Photosynthetica.

[29] Strasser R.J., Tsimilli-Michael M., Srivastava A., Papageorgiou G.C., Govindjee (2004). Analysis of the chlorophyll a fluorescence transient. Chlorophyll Fluorescence: A Signature of Photosynthesis.

[30] Stirbet A., Govindjee (2011). On the relation between the Kautsky effect (chlorophyll a fluorescence induction) and Photosystem II: Basics and applications of the OJIP fluorescence transient. J. Photochem. Photobiol. B.

[31] Suganami M., Konno S., Maruhashi R., Takagi D., Tazoe Y., Wada S., Yamamoto H., Shikanai T., Ishida H., Suzuki Y. (2022). Expression of flavodiiron protein rescues defects in electron transport around PSI resulting from overproduction of Rubisco activase in rice. J. Exp. Bot..

[32] Niyogi K.K., Grossman A.R., Björkman O. (1998). Arabidopsis mutants define a central role for the xanthophyll cycle in the regulation of photosynthetic energy conversion. Plant Cell.

[33] Chen W., Jia B., Chen J., Feng Y., Li Y., Chen M., Liu H., Yin Z. (2021). Effects of different planting densities on photosynthesis in maize determined via prompt fluorescence, delayed Fluorescence and P700 signals. Plants.

[34] Schreiber U. (2004). Pulse–Amplitude–Modulation (PAM) fluorometry and saturation pulse method: An overview. Chlorophyll a Fluorescence a Signature of Photosynthesis.

[35] Yamori W., Masumoto C., Fukayama H., Makino A. (2012). Rubisco activase is a key regulator of non–steady–state photosynthesis at any leaf temperature and, to a lesser extent, of steady–state photosynthesis at high temperature. Plant J. Cell Mol. Biol..

