# Supplementary material for: Effects of OsRCA Overexpression on Rubisco Activation State and Photosynthesis in Maize
Source: Plants (Basel). 2023 Apr 11;12(8):1614. doi: 10.3390/plants12081614 (PMC10142437; doi:10.3390/plants12081614)
Supplement: Supplementary file 1 [file plants-12-01614-s001.zip › plants-2278479-supplementary.pdf]

```
OsRCAα     ..MAAAFSSTVGAPASTPTN..FLGKKLKK.QVTSAVNYHGKSSNIN.RFKVMA.KELDEGKQTDQDRWKGLAYDISDDQ     73
ZmRCAα     MAAAAAFSSTSVVAALAASG..FLGKKLVT.SIIIRNKKKGYDSLHG.ATTTIRVRAMAVNKETEQDRWRGLALDTSDDQ     76
OsRCAβ     ..MAAAFSSTVGAPASTPTN..FLGKKLKK.QVTSAVNYHGKSSNIN.RFKVMA.KELDEGKQTDQDRWKGLAYDISDDQ     73
ZmRCAβ     ..MAAAFSSTVGAPASTPTRSSFLGKKLNKPQVSAAVTYHGKSSSSNSRFKAMAAKEVDETKQTDEDRWKGLAYDISDDQ     78
Consensus     aaafsst    a       flgkkl            g  s                 k t  drw gla d sddq

OsRCAα     QDITRGKGFVDSLFQAPTGDGTHEAVLSSYEYLSQGLRTYDFDNTMGGFYIAPAFMDKLVVHISKNFMTLPNIKVPLILG     153
ZmRCAα     QDITRGKGRVDPLFQAPMGDGTHVAVLSSYDYISQGLRHYSLDNMMDGYYIAPAFMDKLVVHIAKNFMPLPNIKVPLILG     156
OsRCAβ     QDITRGKGFVDSLFQAPTGDGTHEAVLSSYEYLSQGLRTYDFDNTMGGFYIAPAFMDKLVVHISKNFMTLPNIKVPLILG     153
ZmRCAβ     QDITRGKGLVDNLFQAPMGDGTHVAVLSSYDYISQGQKSYNFDNMMDGFYIAKGFMDKLVVHLSKNFMTLPNIKVPLILG     158
Consensus  qditrgkg vd lfqap gdgth avlssy y sqg   y  dn m g yia  fmdklvvh  knfm lpnikvplilg

OsRCAα     IWGGKGQGKSFQCELVFAKMGINPIMMSAGELESGNAGEPAKLIRQRYREAADIIKKGKMCCLFINDLDAGAGRMGGTTQ     233
ZmRCAα     IWGGKGQGKSFQCELVLAKMGINPIVMSAGELESGNAGEPAKLIRQRYREAADMIKKGKMCVLFINDLDAGAGRMGGTTQ     236
OsRCAβ     IWGGKGQGKSFQCELVFAKMGINPIMMSAGELESGNAGEPAKLIRQRYREAADIIKKGKMCCLFINDLDAGAGRMGGTTQ     233
ZmRCAβ     IWGGKGQGKSFQCELVFAKMGITPIMMSAGELESGNAGEPAKLIRQRYREASDLIKKGKMSCLFINDLDAGAGRMGGTTQ     238
Consensus  iwggkgqgksfqcelv akmgi pi msagelesgnagepaklirqryrea d ikkgkm  lfindldagagrmggttq

OsRCAα     YTVNNQMVNATLMNIADNPTNVQLPGMYNKEDNPRVPIIVTGNDFSTLYAPLIRDGRMEKFYWAPTRDDRVGVCKGIFRT     313
ZmRCAα     YTVNNQMVNATLMNIADNPTNVQLPGMYNKEENPRVPIIVTGNDFSTLYAPLIRDGRMDKFYWAPTREDRVGVCKGIFRS     316
OsRCAβ     YTVNNQMVNATLMNIADNPTNVQLPGMYNKEDNPRVPIIVTGNDFSTLYAPLIRDGRMEKFYWAPTRDDRVGVCKGIFRT     313
ZmRCAβ     YTVNNQMVNATLMNIADNPTNVQLPGMYNKEDNPRVPIIVTGNDFSTLYAPLIRDGRMEKFYWAPTREDRIGVCKGIFRT     318
Consensus  ytvnnqmvnatlmniadnptnvqlpgmynke nprvpiivtgndfstlyaplirdgrm kfywaptr dr gvckgifr

OsRCAα     DNVPDEDIVKIVDSFPGQSIDFFGALRARVYDDEVRKWVSDTGVENIGKRLVNSREGPPEFEQPKMTIEKLMEYGYMLVK     393
ZmRCAα     DGVPDEDVVRLVDAFPGQSIDFFGALRARVYDDEVRRWVAETGVENIARRLVNSKEGPPTFEQPRMTLDKLMEYGRMLVE     396
OsRCAβ     DNVPDEDIVKIVDSFPGQSIDFFGALRARVYDDEVRKWVSDTGVENIGKRLVNSREGPPEFEQPKMTIEKLMEYGYMLVK     393
ZmRCAβ     DGVDEEHVVQLVDTFPGQSIDFFGALRARVYDDEVRRWVSETGVENIARKLVNSKEGPPTFEQPKITIEKLLEYGHMLVA     398
Consensus  d v  e  v  vd fpgqsidffgalrarvyddevr wv  tgveni   lvns egpp feqp  t  kl eyg mlv

OsRCAα     EQENVKRVQLAEQYLSEAALGDANSDAMKTGSFYGQGAQQAGNLPVPEGCTDPVAKNFDPTARSDDGSCLYT     465
ZmRCAα     EQENVKRVQLADKYLSEAALGDANDDDLYGKAAQHVRVP.....VPEGCTDPKAGNFDPTARSDDGSCVYN     463
OsRCAβ     EQENVKRVQLAEQYLSEAALGDANSDAMKTGSFYGSAPSS................................     433
ZmRCAβ     EQENVKRVQLADKYLNEAALGEANEDAMKTGSFFK.....................................     433
Consensus  eqenvkrvqla  yl eaalg an d    g
```

Figure S1. Allinment of amino acid sequence of Rubisco activase in maize and rice. White characters on a black background shows the amino acids conserved among all small and large subunits. Grey characters on a black background indicates the amino acids consrved more than three of four subunits. The arrows indicate the important amino acids that interact with Rubisco. Gene bank accession numbers: OsRCAα: BAA97583.1; ZmRCAα: NP001168488.1; OsRCAβ, BAA97584.1 ; ZmRCAβ, BAJ41042.1.

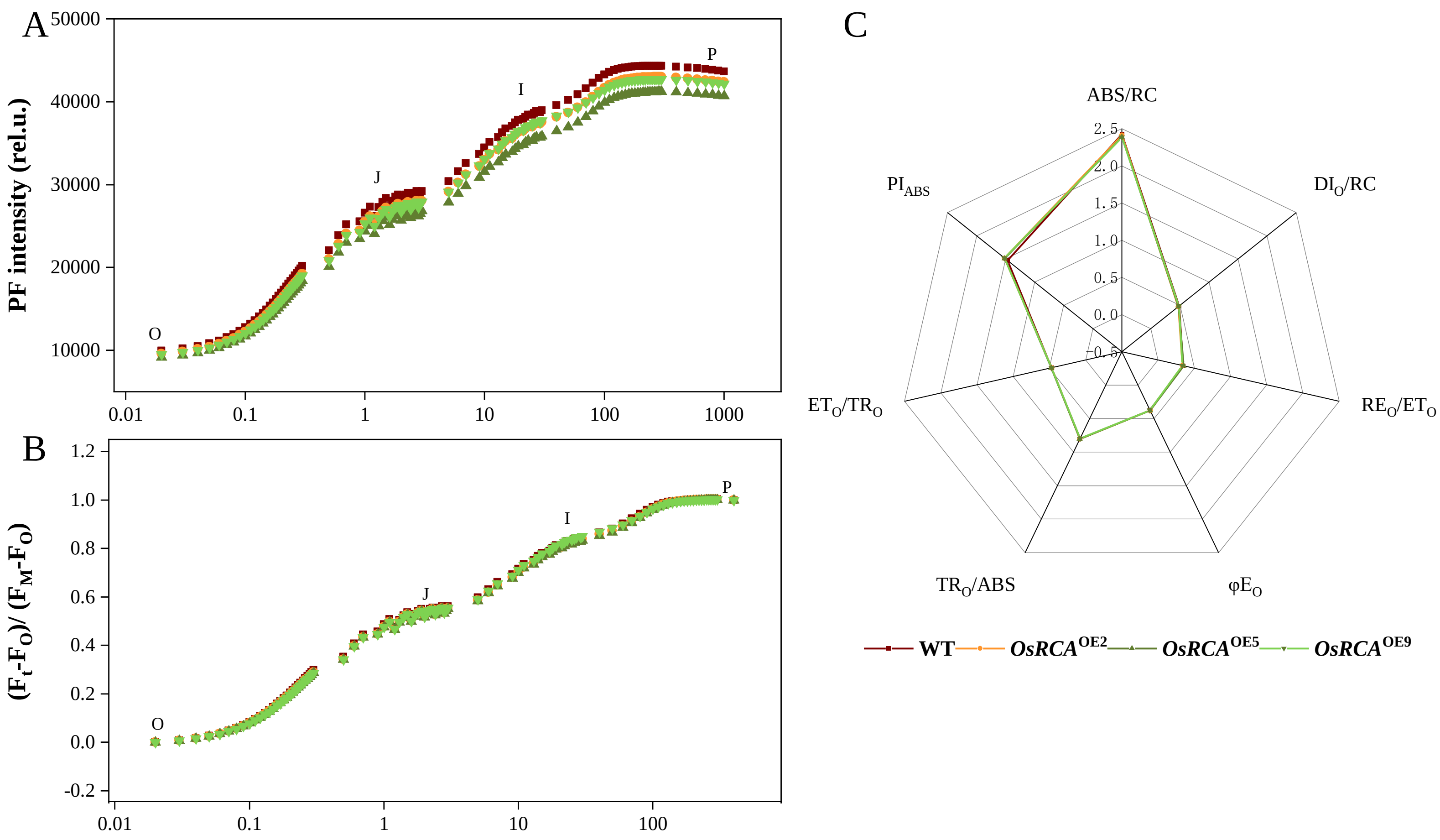

Figure S2. OJIP in WT and *OsRCA*^{OE} lines. (A) Original OJIP curve. Signals are plotted using a logarithmic time scale. (B) O-P standardized curve; the formula is WOP = (Ft - F_O)/ (FP - F_O). Signals are plotted using a logarithmic time scale. PF, prompt chlorphyll a fluorescence. (C) Parameters deduced from JIP test method of WT and *OsRCA*^{OE} lines. ABS/RC: Absorption flux per reaction center (apparent antenna size of an active reaction center); RE_O/ET_O: Efficiency of an electron beyond Q_A^- reducing PSI acceptors; DI_O/RC: Energy dissipated per unit reaction center (t = 0); TR_O/ABS: Ratio of captured light energy to absorbed light energy; φE_O: Quantum yield of electron transport (at t = 0); ET_O/TR_O: Efficiency of electron movement beyond Q_A^-; PI_{ABS}: Performance index on an absorption basis.

Table S1. Primer sequences for PCR

Primer name	Primer sequence (5' to 3')
OsRCA-Forward	CGGAGCTAGCTCTAGAATGGCTGCTGCCTTCTCCTC
OsRCA-Reverse	TGCTCACCATGGATCCAAAGGTGTAAAGGCAGCTGC

Table S2. Primer sequences for qRT-PCR

Primer name	Forward primer	Reverse primer
GAPDH-qPCR	GTCACCGTCTTTGGCATCAG	AGCAGCCTTGTCCTTGTCAGT
OsRCA-qPCR	CCGTGACGGGCGTATGGAGAAG	GCACGAAGAGCGCCGAAGAAATC
